# Personalized Diagnosis in Differentiated Thyroid Cancers by Molecular and Functional Imaging Biomarkers: Present and Future

**DOI:** 10.3390/diagnostics12040944

**Published:** 2022-04-10

**Authors:** Laura Teodoriu, Letitia Leustean, Maria-Christina Ungureanu, Stefana Bilha, Irena Grierosu, Mioara Matei, Cristina Preda, Cipriana Stefanescu

**Affiliations:** 1 Endocrinology Department, “Grigore T. Popa” University of Medicine and Pharmacy, 700111 Iasi, Romania; laurateodoriu@gmail.com (L.T.); maria.ungureanu@umfiasi.ro (M.-C.U.); stefana.bilha@umfiasi.ro (S.B.); cristina.preda@umfiasi.ro (C.P.); 2 Biophysics and Medical Physics—Nuclear Medicine Department, “Grigore T. Popa” University of Medicine and Pharmacy, 700111 Iasi, Romania; irena.raileanu@umfiasi.ro (I.G.); cipriana.stefanescu@umfiasi.ro (C.S.); 3 Preventive Medicine and Interdisciplinarity Department, “Grigore T. Popa” University of Medicine and Pharmacy, 700111 Iasi, Romania; mioara.matei@umfiasi.ro

**Keywords:** thyroid carcinoma, SPECT, PET, molecular biomarkers, artificial intelligence

## Abstract

Personalized diagnosis can save unnecessary thyroid surgeries, in cases of indeterminate thyroid nodules, when clinicians tend to aggressively treat all these patients. Personalized diagnosis benefits from a combination of imagery and molecular biomarkers, as well as artificial intelligence algorithms, which are used more and more in our timeline. Functional imaging diagnosis such as SPECT, PET, or fused images (SPECT/CT, PET/CT, PET/MRI), is exploited at maximum in thyroid nodules, with a long history in the past and a bright future with many suitable radiotracers that could properly contribute to diagnosing malignancy in thyroid nodules. In this way, patients will be spared surgery complications, and apparently more expensive diagnostic workouts will financially compensate each patient and also the healthcare system. In this review we will summarize essential available diagnostic tools for malignant and benignant thyroid nodules, beginning with functional imaging, molecular analysis, and combinations of these two and other future strategies, including AI or NIS targeted gene therapy for thyroid carcinoma diagnosis and treatment as well.

## 1. Introduction

As reported by the American Cancer Society, it is estimated that in 2022 about 43,800 new cases of thyroid cancer (11,860 in men and 31,940 in women) will be found in the United States (US) [1], data that support, once again, the continuing increase in the overall incidence of thyroid cancer. For papillary thyroid carcinoma (PTC), the 5-year relative survival rate in the US, from 2010 to 2016, was near 100% for localized tumors, 99% for regional affection, and 76% for distant metastasis [2]. The International Agency for Research of Cancer (Global Cancer Observatory—Globocan) found that in 2020 thyroid carcinoma was 15th place in Europe for newly diagnosed patients, and 27th place for deaths causes by thyroid carcinoma. Rank was made for all cancer sites [3]. Central and Eastern Europe had in 2020, according to Globocan, 30,288 new thyroid carcinomas cases and 2523 deaths, numbers estimated for both sexes [4]. Central and Eastern Europe had most of the new cases and deaths numbers of thyroid carcinomas than all European regions, in possible relation to the vicinity of Chernobyl [5].

In this battle for prompt diagnosis, two main candidates are involved: imaging (structural and functional) and molecular biomarkers. In this review, we will discuss functional imaging and molecular biomarker diagnosis in thyroid carcinoma: the most important already known facts, what we mainly use in the present time, and future possibilities including artificial intelligence (AI). 

Thyroid nodules are initially explored by hormonal tests, thyroid ultrasound, and fine-needle aspiration cytology (FNAC) to complete the ultrasound features that might suggest a benign diagnosis or a malignant result. Functional imaging, with its rapid progress, including single-photon emission computed tomography (SPECT), positron emission tomography (PET), or fused images combining resolutive structural imaging computed tomography (CT) or magnetic resonance imaging (MRI) in SPECT/CT, PET/CT or even PET/MRI, had and will have a major role in functional assessment of thyroid nodules. Thyroid images will guide clinicians to perform FNAC in order to surgically treat the nodules. Scintigraphy also plays a major role in indeterminate thyroid cytology (Bethesda III–IV) results. Combined with molecular determinations, proper diagnosis is more tangible [6].

**Figure 1:**
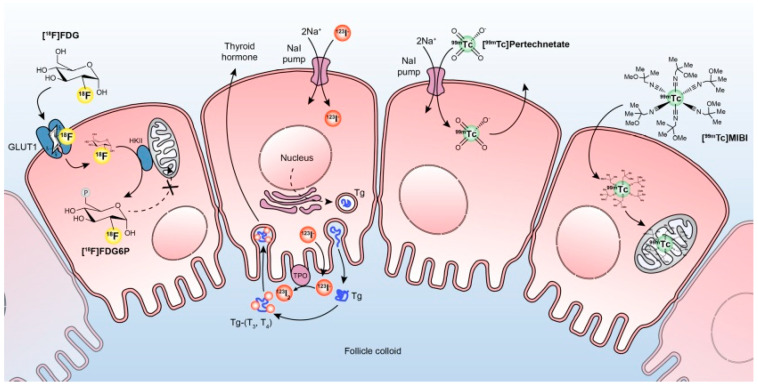
The different metabolic pathways in radioisotope imaging of thyroid nodules. ^18^F FDG is transported into the follicular cell by the transmembrane glucose transporter GLUT1. The intracellular ^18^F FDG is then phosphorylated by HKII to ^18^F FDG6P, which traps the compound and prevents its efflux. In malignant cells, both the expression of GLUT1 transporters and the activity of hexokinase may be increased. Thyroid follicular cells trap ^123^I using the sodium-iodine symporter, which concentrates iodine intracellular and incorporates the ^123^I into thyroglobulin (Tg). The ^123^I is oxidized by thyroid peroxides’, at the follicular cell colloid interface, to neutral iodine, which binds to tyrosine residues on Tg to form thyroid hormones (T3 and T4) stored in the colloid follicular lumen. A similar mechanism is encountered for other iodine radiotracers like ^124^I and ^131^I. ^99m^TcO_4_^−^ is trapped by the thyroid follicular cells in an identical manner as ^123^I, but is neither organified nor incorporated into thyroid hormones and then is not retained in the thyroid. ^99m^Tc MIBI is a lipophilic monovalent cationic agent that crosses the cell membrane and concentrates in the mitochondria due to its positive electric potential. Adapted from Rager O et al. Gland Surg 2019 [6]. Copyright ID: 1187094-1, Nancy International Ltd. Subsidiary AME Publishing Company.

Dedicated radiotracers help clinicians to decide if Bethesda III–IV cytology needs surgical treatment or not. The most frequently used thyroid radiotracers for diagnostic purpose are Iodine-123 (^123^I), Technetium-99m pertechnetate (^99m^TcO_4_^−^), Technetium-99m-methoxy-isobutyl-isonitrile (^99m^Tc MIBI), and Fluorine-18-fluorodeoxyglucose (^18^F FDG). Their uptake mechanism is depicted in Figure 1.

## 2. Functional Imaging Available in Thyroid Carcinomas

Because iodine plays a major role in the physiology and pathophysiology of the thyroid gland, iodine, or iodine analogs (NIS-targeting radiopharmaceuticals) are well suited for thyroid imaging and radioiodine uptake (RAIU) study [7]. Iodine radiotracers, both SPECT and PET, take advantage of sodium-iodine symporter (NIS) to accumulate in the thyroid follicular cells. This quality of those is also exploited in differential thyroid carcinomas (DTC) where radioactive iodine treatment is needed. NIS transports iodine and other substrates as perchlorate and chlorate. In thyroid carcinoma, expression of NIS is decreased due to damage to the DNA by ionizing radiation, decreased expression of SCL5A5, and/or diminished membrane targeting, but is still maintained for imaging and therapeutical purpose by preserving iodine uptake [8].

For diagnostic purposes, **^131^I** was first used about 72 years ago and for therapeutical purposes even sooner [9]. Along with the technological improvement, **^124^I** and **^123^I** come into existence but they need to be generated by a cyclotron, thereby they become more expensive and less available. To prevent energy and money consumption, other NIS-targeted radioisotopes were developed. 

**The ^131^I scintigraphy** is used after radioiodine treatment because of the inexpensive radioisotope production that transforms it into the most available theragnostic radiotracer. It uses low radioactivity and that might be a problem for some thyroid lesions because it will not capture a clear image after ^131^I administration on planar images or SPECT. This is the moment when other tracers must be taken into consideration and ^124^I is one of them [8]. More recently, ^131^I SPECT/CT has dramatically improved WBS image interpretation. The procedure has a better contrast resolution than planar acquisition and also obtains better anatomic fusion images. SPECT/CT proved capable of improving the performance of planar WBS in detecting radioiodine avid foci, increasing sensitivity and accuracy, and allowing precise anatomic localization and characterization of the lesions, improving DTC staging, and subsequent patient management [10].

^123^I is an ideal isotope for the assessment of thyroid function due to its low radiation burden resulting in an effective dose of 0.2 mSv/MBq and revealing NIS function, as well as organification. It has a longer physical half-life of 13 h, and usually, images are taken at 2−6 h and 24 h after oral ^123^I administration, allowing for repeated uptake measurements and/or imaging until 30 h. However, it is less available and slightly more expensive than **^99m^TcO_4_^−^** and requires a specific order [11]. 

**^124^I** behaves similarly to ^131^I and has a 4.2-day half-life which makes it suitable for detecting recurrence of DTC or metastasis. Given the added benefit of PET/CT with ^124^I, radiotracer uptake can be detected in some regions that do not have an evident structural correlation with CT [12].

A systematic review compared the diagnostic power of ^124^I over ^131^I whole-body scan (WBS) and found that ^124^I images are superior to ^131^I WBS or even to post therapeutically scans. Low diagnostic activities of ^124^I PET have fewer side effects than a diagnostic 1.11 GBq (30 mCi) exploratory scans or the post-therapy scans obtained after higher therapeutic activities of ^131^I. The radiation exposure of 5–10 mSv from the administration of 50–100 MBq (1.4–2.7 mCi) of ^124^I compares favorably with the 60 mSv after receiving 1000 MBq (27 mCi) of ^131^I [13].

However, there are some flaws in using ^124^I for diagnostic images including its relatively high cost and complex decay schema, which may lead to background noise and voxel oversaturation even at low administered activities [12].

**^99m^Tc pertechnetate (^99m^TcO_4_^−^)** is one of the radioisotopes that are less expensive, which is why its availability is increased. ^99m^TcO_4_^−^ is a pharmacologic mimic of iodine which is concentrated within the thyroid cells by the NIS transport system; however, it is not organified and therefore washout from thyroid cells occurs after 30 min of radiotracer administration. Although the thyroid gland does not organize ^99m^TcO_4_^−^ in the majority of cases, the uptake, and imaging data provide sufficient information for the accurate functional diagnosis of thyroid conditions including nodules. However, in some cases, the appearance of a thyroid nodule may be discordant on radioiodine and pertechnetate scans due to iodide organification defects in the nodule that results in a rapid washout of radioiodine (so-called trapping only nodule) [7]. There is a similarity between the general characteristics of ^99m^TcO_4_^−^ uptake and the transport of radioactive iodine, which is why ^99m^TcO_4_^−^ represents a substrate for NIS, as well as ^123^I/^131^I. This analogy is due to its negative electric charge as well as iodine and a molecular weight > 100u [14]. It was demonstrated that the affinity of ^99m^TcO_4_^−^ for NIS is even greater than iodine and is not incorporated into thyroglobulin therefore not retained in the thyroid. ^99m^TcO_4_^−^ is routinely used for thyroid nodules and DTC imaging. It emits no β rays but 140 keV gamma rays, which is an ideal peak for gamma camera imaging as well as its suitable half-life (6 h) [14,15].

Other radioisotopes that use NIS are still in research for PET scanning as tetrafluoroborate (^18^F TFB). Studies have shown in vivo biodistribution of PET scanning after 60 min injection and the obtained thyroid images indicated that ^18^F TFB can be rapidly concentrated into NIS expressing tissues and could be used as a potential diagnostic tool [16]. It was first born in 1950, then reborn in 2010, and it was first used for PET scanning on animals in 2015, then in 2017 for human purposes [17].

^18^F TFB is retained but not organified by the thyroid. ^18^F TFB may benefit from having no need for thyroid-stimulating hormone (TSH) stimulation in detecting DTC metastases after the surgery. The thyroid remnant tissue after ^131^I ablation could be detected by ^18^F TFB complementary to ^131^I, after the radioiodine therapy. ^18^F TFB delivers a much lower absorbed radiation dose than the radioiodine family of tracers for both medium and high levels of thyroid uptake according to O’Doherty J et al. [18]. 

Research has found that this radiotracer is well tolerated by patients and no adverse effects were noted. Its diagnostic and therapeutic purpose is extremely desired because it acts over NIS, found predominantly in thyroid and breast tissue. This mechanism helps patients get not only a specific diagnosis by PET scanning but also a theragnostic response by facilitating thyroid and breast carcinoma treatment over NIS gene therapies. ^18^F TFB PET will enable quantitative estimation of NIS activity and, with this particularity, patients will benefit from intense monitoring of disease progression and major therapy response [19].

Recent studies compared ^18^F TFB PET with ^131^I scintigraphy WBS and ^18^F FDG PET/CT to conclude if the first two radioisotopes have better sensitivity than the last one. Dittmann M et al. compared the power of detection in recurrent DTC of these three tracers. A failure in detecting NIS expressing local recurrences or metastases of DTC might result both in a missed opportunity for localized therapy and an erroneously assumed TENIS (thyroglobulin elevated negative iodine scintigraphy) syndrome found in cases of recurrent DTC with dedifferentiation of tumor cells that decrease NIS expression but increases ^18^F FDG metabolism, in turn. In contrast to iodine nuclides, ^18^F TFB can easily be synthesized by a cyclotron and offers a favorable half-life, dosage, and biodistribution, as well as superior PET image quality. ^18^F TFB PET had 52% sensitivity and accuracy, slightly increased than 47.6% of ^18^F FDG PET/CT. The combination of these two PET tracers improves sensitivity and accuracy by over 64% with a 100% positive predictive value (PPV). Following their mechanism, ^18^F TFB, using NIS expression, and ^18^F FDG-6-phosphate that cannot proceed down the glycolytic pathway and therefore accumulates in cancerous cells or infiltrating immune cells could detect recurrence of DTC even if NIS expression is lost due to dedifferentiation [6,20].

**Figure 2:**
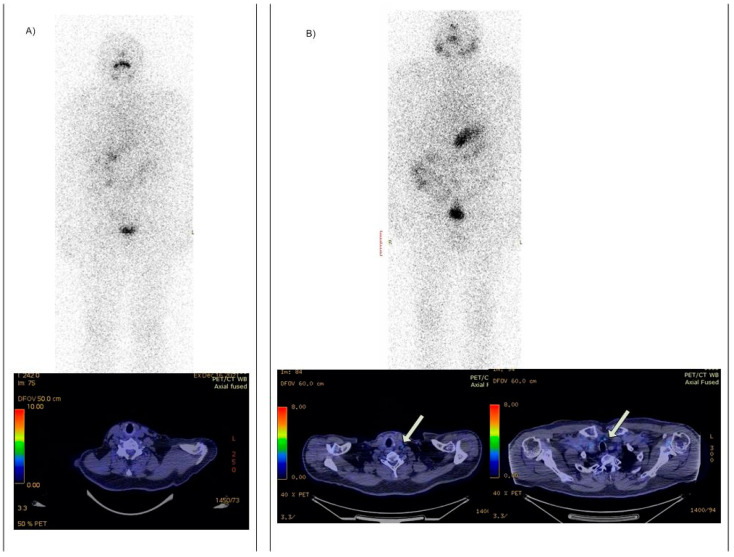
Discordant vs. concordant TENIS patterns in two different patients from ROI Iasi; (**A**) negative WBS with negative ^18^F FDG PET/CT in an 80-year-old female patient with papillary carcinoma resected in 2010 and radioiodine treated, with a total of 15.91 GBq (430 mCi), with rising Thyroglobulin values over last two years (18 ng/mL); (**B**) negative WBS in a 68-year-old male patient with papillary carcinoma resected in 2019, radioiodine treated with 6.3 GBq (170 mCi), had persistently increased thyroglobulin (10 ng/mL) and positive ^18^F FDG PET/CT for lateral (**left** arrow) and mediastinal (**right** arrow) lymph nodes.

Unpublished data from Regional Oncology Institute (ROI), Iasi-Romania, relates patients with TENIS pattern with recurrent DTC (increased thyroglobulin or cervical ultrasound masses), negative WBS, and negative ^18^F FDG PET/CT (Figure 2). Such patients could benefit from functional images like ^18^F TFB PET. 

**^18^F FDG** is using transmembrane glucose transporters in the same manner as naturally glucose molecules. The intracellular ^18^F FDG is then phosphorylated by hexokinase to ^18^F FDG-6-phosphate, which traps the compound and prevents its efflux. In DTC malignant cells, both the expression of GLUT1 transporters and the activity of hexokinase may be increased; therefore, ^18^F FDG accumulates in malignant cells [6].

Many studies explored in what particular condition ^18^F FDG is compatible with proper diagnosis in thyroid nodules, or if this radiotracer could differentiate potential malignant nodules by a simple scan [13]. Guidelines recommend ^18^F FDG scans only in patients with thyroid carcinomas, especially DTC, which are suspect of recurrence (increased thyroglobulin and negative iodine WBS) [13]. 

Covering the above debate, meta-analyses were published finding answers about the risk of malignancy (ROM) in thyroid incidentalomas found on ^18^F FDG PET/CT results (Table 1), other studies show how precise could ^18^F FDG PET/CT be in discovering malignant nodules in cases of Bethesda III–IV (Table 2) or how accurate could this radiotracer be in cases of DTC restaging (Table 3).

The median ROM of thyroid incidentalomas identified over ^18^F FDG PET and PET/CT images, from the above meta-analyses, was 25%. It is visible that Leijer et al. had the lowest ROM (12%) probably because of the large population included in this study. 

For the indeterminant nodules, the median prevalence of malignancy was 25%, and Qichang et al. obtained the best PPV and specificity from all selected meta-analyses. 

^18^F FDG PET/CT is a great tool in restaging DTC, especially in TENIS syndrome cases. Specificity for ^18^F FDG was about 80% in selected meta-analyses, and Qichang et al., including the largest number of patients, reached the highest specificity of 84%.

**Figure 3:**
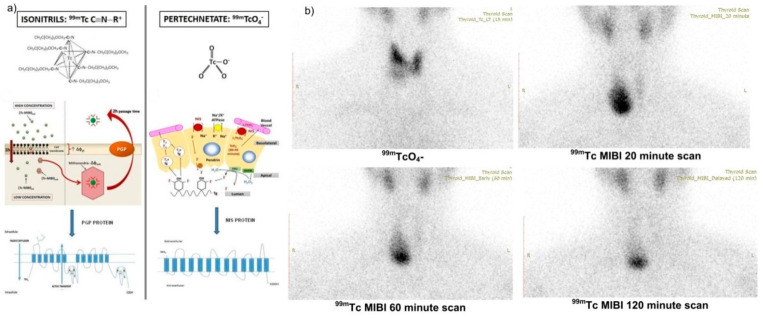
Different radiotracer’s uptake mechanisms of thyroid nodule of a 42-year-old female patient. (**a**) Depicted ^99m^TcO_4_^−^ and ^99m^Tc MIBI mechanism for thyroid cells [14]; (**b**) decreased ^99m^TcO_4_^−^ uptake in right thyroid nodule with increased ^99m^Tc MIBI uptake in the same nodule with reduced efflux of the tracer in late images, including 60 min scan, which carries a great risk for malignancy (unpublished data of ROI Iasi-Romania).

**^99m^Tc MIBI** is a lipophilic monovalent cationic complex that crosses the cell membrane reversibly, but then it is trapped within the mitochondria due to its electric potential. Hyperfunction and active cell growth phase (of the cell cycle) of thyroid and parathyroid cells lead to the uptake of higher amounts of the radiotracer. Thyroid nodules that uptake and do not liberate ^99m^Tc MIBI at one hour, reflect actively functioning mitochondria, and therefore cellular oxidative metabolism as seen in Figure 3 [6,7,14].

^99m^Tc MIBI images are interpreted visually and findings include a low, an isointense, or an increased radiotracer accumulation in the thyroid nodule in comparison to the normal thyroid tissue and in comparison to pertechnetate thyroid images. A “mismatch” describes a hypofunctioning thyroid nodule on ^99m^TcO_4_^−^ scintigraphy and increased uptake of ^99m^Tc MIBI in comparison to the uptake of the adjacent thyroid tissue [7].

Treglia et al. evaluated the diagnostic value of ^99m^Tc MIBI for thyroid nodules in a meta-analysis (21 studies with a total of 2000 patients) which demonstrated a good sensitivity but low specificity of this radiotracer. This meta-analysis opened up new diagnosis opportunities in order to assess the prevalence of malignant thyroid nodules by ^99m^Tc MIBI scintigraphy [42].

Comparative studies of hypofunctional nodules, further investigated by ^99m^Tc MIBI scan, and their ultrasound characteristics, showed clear superiority of NPV (100% vs. 91%) and regarding ^99m^Tc MIBI, this tracer had a sensitivity of 100% compared to 87% of the ultrasound [42].

The ^99m^Tc MIBI semi-quantitative scintigraphy method, proposed by Erdil TY et al. [43], calculated the index capture of the radiotracer (RI), from hyperfunctional nodules, using the next formula with some great positive results (sensitivity 95.2%, specificity 89.4%, and accuracy 92.5%):

Mean nodular ^99m^Tc MIBI uptake (early scan) − mean background ^99m^Tc MIBI uptake (early scan) = early result (ER);

Mean nodular ^99m^Tc MIBI uptake (late scan) − mean background ^99m^Tc MIBI uptake (late scan) = late result (LR);

LR/ER × 100 − 100 = washout index (WOind);

(LR − ER) × 100/ER= RI

There was a marked superiority in the assessment of the ROM in thyroid nodules compared to the result of the late images ^99m^Tc MIBI, as it was known until then [43,44].

Saggiorato et al. used the semi-quantitative method, with high specificity and sensitivity, in cases of follicular lesions, and the uptake index (RI) managed to differentiate the nodules by benignity versus malignancy. The same study issued molecular biomarkers from FNAC for the most accurate indications of surgical therapeutic conduct [45].

This investigation also has weaknesses such as the calculated washout being human dependent, as the region of interest (ROI) is manually delimited. This weak point can be overcome by gaining the necessary experience in investigating as many patients as possible, thus limiting redundant surgeries. Evaluation of thyroid nodules by ^99m^Tc MIBI has grown in recent years, but a protocol for a specific acquisition of this radiotracer is not established, so cohort studies must be performed to determine the diagnostic value of ^99m^Tc MIBI. 

**^68^Gallium (^68^Ga) prostate-specific membrane antigen (PSMA)**, based on PET/CT or PET/MRI, is a whole-body imaging technique, currently performed for the detection of prostate cancer (PCa) lesions. PSMA is a type II transmembrane glycoprotein receptor with glutamate carboxypeptidase/folate hydrolase activity and is expressed in the normal prostate secretory epithelium and highly expressed in PCa. PSMA has been found to be expressed in other solid tumors such as gastric, colon, breast, lung, adrenal, bladder, renal cell carcinoma, and, recently, thyroid carcinoma. 

^68^Ga PSMA has the capability to detect thyroid lesions, as demonstrated in a systematic review which concluded that thyroid incidentalomas found on this radiotracer have some potential of being primary thyroid carcinomas. Of 23 thyroid detected incidentalomas, 5 were primary thyroid carcinomas (4 papillary thyroid carcinoma, 1 follicular thyroid carcinoma-FTC) [46]. A recent study found that the incidence of thyroid incidentalomas was only 1.1% in patients monitored for PCa between 2016 and 2021. Histological confirmation was possible for two patients, one had benign thyroid adenoma and the other had intrathyroid renal cell metastasis [47].

Another study found that PSMA thyroid incidentalomas could predict a primary thyroid carcinoma in 4% of 341 patients included in this study, and of them, 2 patients (15%) had DTC [48].

A feasibility research study found that ^68^Ga PSMA PET/CT improves DTC diagnosis in some particular cases compared to ^18^F FDG PET/CT. In some cases for which ^18^F FDG PET is not useful due to very low uptake, ^68^Ga PSMA PET could serve as a more sensitive diagnostic PET agent. The exploration of ^68^Ga PSMA PET as a possible theragnostic agent in thyroid cancer could be explored in DTC [49]. 

This supposition is further supported by a study that compared ^68^Ga PSMA and the classic DTC diagnostic tool (^131^I SPECT) and found that ^68^Ga PSMA is more accurate than ^131^I in detecting more metastatic lesions, which could be related to the expression of type II carboxypeptidase in the vascular endothelium. 

The benefit of ^68^Ga PSMA is the independence of NIS, so the patients will not need TSH stimulation for the investigation. Another benefit found by recent studies is that thyroid lesions found on PSMA will bring a poor prognosis of aggressiveness, and could predict radioiodine refractory disease [50].

Another PSMA imaging agent, ^18^F DCFPyl(2-(3-{1-carboxy-5-[(6-[(18)F]fluoro-pyridine-3-carbonyl)-amino]-pentyl}-ureido)-pentanedioic acid), was recently developed and, in a prospective study involving this tracer in DTC lesions, showed significantly increased neovascular PSMA expression in lymph nodes of DTC as well as the ability of ^18^F DCFPyL to detect lesions that were previously unnoticed by RAI based imaging as well as ^18^F FDG PET/CT [51].

**^18^F choline PET/CT** is another radiotracer that uses as vector molecule choline, a precursor in the biosynthesis of phosphatidylcholine. It has been demonstrated to be very useful in the evaluation of patients affected by PCa, but it also seems to be useful in thyroid carcinomas. An increased cell membrane choline metabolism is not specific to PCa, in fact, it has been demonstrated that it can also be increased in other pathological conditions, both oncological and non-oncological. Albano D et al. found that ^18^F choline PET/CT shows evidence of focal thyroid incidentaloma and lately discovered that approximately one-fourth of focal thyroid uptakes are malignant [52].

However, it must be taken into consideration that ^68^Ga PSMA and choline radiotracers are mainly used in prostate cancer, so women, who have the most frequent thyroid lesions, are excluded from the analysis. 

## 3. The Future in Theragnostic: DTC Radionuclide Somatostatin Receptor and Redifferentiation Therapies

The involvement of the somatostatin receptor (SSTR) in the regulation and proliferation of normal thyroid cells and tumor tissue was firstly mentioned in 1990 [53] and demonstrated by ^111^In somatostatin analog scintigraphy in 1995 [54]. Following these findings, several studies used different radiolabeled somatostatin analogs for diagnosis and/or treatment, respectively theragnostic approach (peptide receptor radionuclide therapy—PRRT after somatostatin teceptor scintigraphy—SRS) of medullary thyroid carcinoma (MTC) and non-radioiodine avid DTC [53]. 

A radiolabeled somatostatin analog generally consists of the following three main parts: a synthetic analog of somatostatin (cyclic octapeptide) such as octreotide, Tyr3-octreotate, or Tyr3-octreotide, a chelator such as DTPA or 1,4,7,10-tetraazacyclododecane-1,4,7,10-tetraacetic acid (DOTA), and a suitable radioactive component. Speaking about thyroid carcinomas diagnosis, the most studied radioisotopes were ^99m^Tc, ^111^In, ^64^Cu, ^89^Zr, ^201^Tl, and ^68^Ga (either alone, or labeling a specific vector molecule) with great involvement in cancer radioimmunoimaging [55]. 

On the other hand, radionuclides commonly used for PRRT of thyroid carcinomas are Lutetium 177 (^177^Lu) and Yttrium 90 (^90^Y). Using radionuclide for theragnostic purposes must be personalized, with a close relationship between tumoral cells’ phenotypic characteristics and physic proprieties of the chosen radionuclide. ^177^Lu emits intermediate energy β particles (133 keV), resulting in a maximum tissue penetration range up to 3 mm, which makes it a preferable radionuclide for smaller tumors. In addition to β particles, ^177^Lu emits two gamma peaks of 113 and 208 keV, which makes it a suitable compound for post-therapeutic gamma camera dosimetry. ^90^Y has the highest energy of β particles (935 keV) and the highest maximum tissue penetration, resulting in a “crossfire effect” on nearby tumor cells, which is why this radionuclide is preferred in bigger tumors, including thyroid carcinomas [53]. 

Regarding the labeled molecule, the research, aims to match the radiotracer with the expressing types of SSTR in the different types of thyroid carcinoma cells. There are reported conflicting results for SSTR subtype expression on different thyroid tumor cells. According to some in vitro studies, SSTR2 is the predominant subtype in DTC tissue. Different SSTR subtypes have been identified in thyroid cancer tissue in vitro. The SSTR2a subtype was expressed in 66% and SSTR2b in 83% of DTC tissue specimens studied with immunohistochemical staining [56]. The commonest receptor expressed in DTC was SSTR5, while in benign disease was SSTR2b. Strongest expressed in benign and malignant thyroid disease was SSTR1 [56]. SSTR2a correlates with the overall survival rate in patients with MTC and is considered a favorable prognostic marker in stage IV MTC patients [57]. 

Klagge et al. investigated mRNA expression of different SSTR subtypes in thyroid cancer cells. Predominant expression of SSTR2 and SSTR5 and a weak expression of SSTR1 and SSTR3 were found in DTC cells. Compared to normal thyroid tissue, SSTR2 and SSTR3 were significantly upregulated in PTC and SSTR5 mRNA expression was increased in both PTC and FTC. However, it must be pointed out that the SSTR are mostly in vitro studies performed on the tissue specimens of uncomplicated cases of DTC [58]. Our daily patients are treated with repeated doses of radioiodine for several years before dedifferentiation occurred. This long process may lead to changes in the SSTR subtypes profile in the DTC cells that we are not aware of, causing a lower affinity of the PRRT [58].

DOTATOC was developed and labeled with different radionuclides for diagnostic imaging as well as for therapy. As a consequence of the replacement of phenylalanine by tyrosine as the third amino acid in the octreotide, this molecule has increased hydrophilicity and affinity for SSTR2. In addition, DOTA is used in this compound as the chelator allows stable binding with ^90^Y or ^177^Lu. The third generation of somatostatin analogs is DOTA-1-Nal3-octreotide (DOTANOC), in which the third amino acid is 1-naphthylalanine instead of phenylalanine. This compound has been shown to improve affinity for SSTR2 and with a higher affinity to SSTR3 and SSTR5. Newer somatostatin analogs such as DOTANOC-ATE ([DOTA-1Nal3, Thr8]-octreotide) and DOTA-BOC-ATE ([DOTA, BzThi3, Thr8]-octreotide) are in development. These “fourth-generation analogs” have been reported to show very high affinity for SSTR2, SSTR3, and SSTR5 and intermediate-high affinity for SSTR4, which possibly make them good candidates for thyroid tumor cells that express high levels of SSTR3 or 5, despite low expression of SSTR2 [53].

Because somatostatin receptors are less expressed over follicular thyroid cells, PRRT-DOTA was less preferred in DTC than in MTC patients. Versari et al. made a prospective study that enrolled progressive DTC radioiodine negative patients, already surgically treated, with no possible surgically reintervention. ^90^Y DOTATOC, used in this study, induced disease control in 7/11 patients: 2 partial remissions and 5 stable diseases with a duration of response ranging from 3.5 to 11.5 months. SSTR was expressed in over 58% of radioiodine negative DTC [59].

A recent systematic review that set out to study the efficacy and safety of PRRT in advanced radioiodine refractory DTC included 41 studies and observed that 25.3% had partial remission and only 10% had complete remission from 157 patients treated with PRRT, which are included in this analysis [60]. 

A meta-analysis observed that patients treated for DTC with ^177^Lu had an objective response rate (ORR) of 24.52% (95% CI, 5.26–56.70%), and, also, these ^177^Lu treated patients had a disease control rate (DCR) of 67.26% (95% CI, 35.42–90.42). For ^90^Y, the pooled proportion of ORR was 13.82% (95% CI, 5.88–26.07%) and DCR was 50.86% (95% CI, 36.76–64.86%). Serious adverse effects were encountered only in 2.82% of patients included in this meta-analysis [61], therefore these data support that PRRT has an important utility in radioiodine refractory DTC with the maximum disease control (67%) and a minimum adverse effect (2.82%).

Other single-center experiences were published, small cohorts (5–8 patients) were included in these studies and the final conclusion was that PRRT does not improve disease outcomes in radioiodine resistant patients, with a progressive biochemical disease being encountered in 62% and imagistic progressions in 75% of the scans. Partial remission was found in 37.5% of biochemical data [62,63].

A new approach in the DTC PRRT field is ^177^Lu PSMA. Studies have shown that PSMA is uptaken in thyroid nodules, so this radiotracer will help patients with refractory thyroid carcinoma to be theragnosticaly approached. One of the two patients who underwent ^177^Lu PSMA therapy showed a slight, temporary response in one study that enrolled 5 patients with refractory DTC [64]. It will be necessary for more prospective studies to determine if ^177^Lu PSMA could be efficient for therapy or not in these particular patients. 

^177^Lu PSMA seems to be more likely effective than ^177^Lu DOTATE treatment, but more prospective studies will be needed to confirm it. Overall, PSMA expression in the neovasculature was observed in PTC (61%—134/220 patients), FTC (56%—43/77), MTC (83%—104/126), and anaplastic thyroid carcinoma (ATC) (63%—12/19). Of the PTC and FTC tumors that became dedifferentiated, neovascular PSMA expression was reported in 63% of all the tumors (15/24) [65]. 

In a study by Assadi et al., a progressing metastatic radioiodine refractory DTC patient was treated with ^177^Lu PSMA. The patient previously received RAI therapy, Sorafenib therapy for 6 months, and PRRT using ^177^Lu DOTATATE (1 cycle, 7.4 GBq). Thereafter, 1 cycle of 7.4 GBq ^177^Lu PSMA was given to the same patient. Post therapy whole-body SPECT imaging revealed higher uptake of ^177^Lu PSMA compared with whole-body SPECT imaging following ^177^Lu DOTATE treatment. PSMA proved to be more likely effective in this patient [66]. As a general conclusion of currently available case reports, it can be stated that thyroid cancer (differentiated and medullary) is one of the most relevant tumors to further be explored by the potential of PSMA PRRT [67].

### NIS Targeted Gene Therapy

The multitude and complexity of NIS regulatory factors bring together plenty of studies to research the therapeutic effects of NIS over progressive radioiodine negative DTC. 

**Figure 4:**
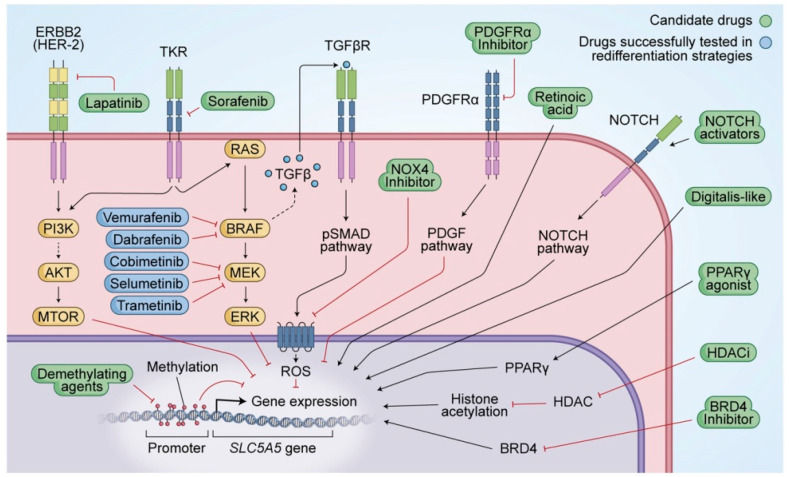
Current and future activators of NIS symporter in treating radioiodine refractory disease: MAPK (mitogen-activated protein kinase) (RAS/RAF/MEK) and Phosphoinositide 3-kinases (PI3K/protein kinase B, AKT/mechanistic target of rapamycin (mTOR)) are key signaling pathways in thyroid cancer pathogenesis. Signaling cascades can be blocked by new targeted therapies. The crosstalk between MAPK and PI3K through RAS represents a tumor escape mechanism from known multi-kinase inhibitors and selective inhibitors of BRAF. The PI3K-AKT pathway activation leads to suppression of NIS glycosylation and surface translocation. The inhibition of mTOR promotes redifferentiation of thyroid cancer cells by upregulation of NIS mRNA and protein expression through increased transcription of thyroid transcription factor 1 (TTF1). Another important positive regulator of NIS expression is Phosphatase and tensin homolog (PTEN). TSH signals through the heterotrimeric G-protein complex and through activation of cyclic adenosine monophosphate (cAMP) increase transcription of the NIS gene. Aberrant activation of the MAPK signaling pathway inhibits Phosphatidylinositol glycan anchor biosynthesis class U protein (PIGU) expression and NIS basolateral transport. Pituitary tumor-transforming gene 1 (PTTG1) and pituitary binding factor (PBF) overexpression results in decreased NIS levels in thyroid cancer. Genetic and epigenetic alterations in the RTK/BRAF/MAPK/ERK and PI3K-AKT-mTOR pathways by acquired point mutations, chromosomal rearrangement, or aberrant gene methylation underlies the diminished NIS signaling central to RAI refractoriness. First, BRAF activates TGFβ-transforming growth factor β)/Smad3 signaling, which directly impairs the ability of the thyroid-specific transcription factor paired box gene 8 (PAX8) to bind the NIS promoter in follicular cells. Second, BRAF epigenetically regulates NIS by driving histone deacetylation of the H3 and H4 lysine residues of the NIS promoter, directly preventing its transcription [68]. Copyright ID 1188004-1, Bioscientifica Limited.

Additionally, studies demonstrated that dedifferentiation is related to a decrease in or a loss of NIS expression, and/or targeting to the plasma membrane, where NIS is fully effective, which results in the loss of iodine uptake in thyroid cells. The concept of redifferentiation strategy has emerged with the purpose to find one or more drugs capable of restoring radioiodine sensitivity for radioiodine refractory thyroid cancers (Figure 4) [68].

NIS expression can be regulated at both transcriptional and posttranslational levels. There are three primary signaling pathways that have been identified. First, activation of transforming growth factor-β (TGF-β) stimulates SMAD3, which inhibits the binding of PAX-8 to the NIS upstream enhancer (NUE). Second, toll-like receptor 4 (TLR4) stimulates nuclear factor kappa-light-chain-enhancer of activated B cells (NF-κB) p65 which activates PAX-8 to bind NUE. Third, PTTG1 and PBF interrupt the interaction between PAX-8 and NUE which suppresses NIS expression. Another crucial factor determining NIS functionality is the translocation of NIS from the cytoplasm to the plasma membrane by posttranslational modification [69].

To complete the signaling pathways in the redifferentiation of thyroid carcinoma, MAPK and PI3K/AKT/mTOR are the key pathways of thyroid cancer pathogenesis. 

Current clinical trials study redifferentiation activators that could bring continuity in radioiodine treatment, as listed by Oh et al. A number of activators were studied:

Dabrafenib (interventional study), the most popular BRAF inhibitor, restored, in 6/10 patients, radioiodine activity with partial remission in 2/6, and stable disease in 4/6 patients [70]. 

Vemurafenib (pilot study), increased radioiodine uptake in 4/10 patients and 3/4 patients had tumor regression after radioiodine therapy [71]. 

Selumetinib (pilot study), also a popular MEK inhibitor, increased iodine activity in 12/20 patients and reached partial remission in 5/8 patients after re-administration of radioiodine treatment [72]. 

Rosiglitazone (pilot study), a peroxisome proliferator-activated receptor gamma (PPARɣ) agonist, increased iodine activity in 5/9 patients with a particularity than the other pilot studies, treatment was continued for 6 months with a higher dose. Tumor regression was found in 3/9 patients after radioiodine treatment [73]. 

Retinoic acids: Bexarotene, Isotretinoin, and Tretinoin (Phase II study) are used to increase activity with very good results in some pilot studies. Overall retinoic acids had a minor improvement in iodine reuptake as concluded by meta-analysis, but compared to tyrosine kinase inhibitors (TKi) adverse effects, might be an alternative solution to determine reactivation of NIS [74]. 

Histone deacetylase inhibitors (HDAC), Valproic acid, or lithium faintly increase iodine uptake, 0/10 for valproic acid and 5/12 with lithium, with no response after radioiodine treatment for lithium 0/12 patients [69]. 

The next step will consist of a combination of different medications, especially those MEK inhibitors. Vemurafenib treatment followed by Dabrafenib increased thyroglobulin, a sign of redifferentiation, with an immediate loss of the effect after discontinuation of TKi [75]. Dabrafenib ± Trametinib, Vemurafenib (BRAF V600E inhibitor) + Trametinib (MEKi) increased RAI uptake by 69% with best responses in RAS mutated patients [76]. Trametinib ± Dabrafenib, Vemurafenib + Cobimetinib (MEKi) obtained 67% of radioiodine uptake and the best responders were BRAF mutated patients [77].

The future is certainly to move toward strategies combining two or more drugs, with a complementary mechanism of action, that is, drugs inhibiting the MAPK pathway output combined with drugs acting on the epigenetic regulation of the NIS and drugs that target the NIS to the plasma membrane, for example [68]. There are still ongoing clinical trials purposing redifferentiation of DTC, selecting patients for Vemurafenib, Dabrafenib, Trametinib, Selumetinib, and Imatinib treatment with promising results for 2023. 

## 4. Molecular Biomarker Diagnosis in Differential Thyroid Carcinoma

PTC is often characterized by RET chromosomal rearrangement, or point mutation of RAS or BRAF proto-oncogenes, all of which are able to trigger the activation of MAPK cascade and therefore the appearance of thyroid carcinoma. Mutations in RET, BRAF, and RAS, when analyzed simultaneously using DNA microarrays, have been shown to generate distinctive expression profiles that can be used as a genetic signature for their accurate classification. Aside from these mutations, the overall differences in the expression of more than 200 other genes between PTC and normal thyroid tissues, when taken together, showed strong potential to be used as a molecular signature (next gene sequencing—NGS) to discriminate the malignancy. MicroRNAs (miRNAs) are small endogenous non-coding RNAs of approximately 22 nucleotides in length. They have key roles in post-transcriptional regulation of genes by repressing translation and/or degrading their messenger RNA targets in the cytosol, as well as in the alteration of gene expression in the nucleus. Their ubiquitous roles in gene regulation require the involvement of miRNAs in many intracellular regulatory processes, such as differentiation, proliferation, and apoptosis. Hence, dysregulation of miRNAs has been associated with many pathological disorders, including thyroid carcinoma [78].

Fine-needle aspiration cytology (FNAC) is the starting point of preoperative diagnosis of thyroid nodules. In a meta-analysis of more than 25,000 FNAC samples of the thyroid gland, for 25% of which the subsequent pathology report was available, the average diagnostic sensitivity of the method was 97%, specificity 50%, diagnostic accuracy 69%, PPV 56%, and the NPV was 96% [79]. The real diagnostic problem in FNAC results consists in Bethesda I—nondiagnostic, when cytology is insufficient for an accurate histological result, III—indeterminate, when histology is not able to determine if modified nucleus are indeed a malignancy pattern or not, and Bethesda IV—suspicious for follicular neoplasm, just a supposition with no strong argument for malignancy diagnosis. For these kinds of results, molecular biomarkers have a major impact in determining the ROM. To rule out ROM in cases of Bethesda III and IV, a meta-analysis discovered an odds ratio of 3.63 (3.06–4.35) [80]. ROM for the Bethesda III category was 5–28% and for Bethesda IV was 15–40% [79,81].

Clinicians will recommend for most patients with indeterminate cytology (including all those belonging to the Bethesda IV category) to do the surgery or molecular testing, but postoperative histological examination shows that 70–80% of thyroid nodules turn out to be benign, and the surgical procedure appears to be unnecessary [82]. To overcome the limitations of the cytological analysis, several molecular tests for preoperative diagnosis of thyroid nodules were developed. Some of these tests involve the detection of somatic point substitutions (BRAF and RAS) and/or translocations (RET-PTC, PAX8-PPARγ). Other approaches involve profiling protein-coding genes or miRNAs or combining analyses of somatic mutations, mRNA, and miRNA levels [82].

In a recent study that included FNAC Bethesda III and IV with a molecular biomarkers diagnosis of benignity, the ROM was 4.8%. Taking into account the cancer prevalence (30% for each of these categories) in the tested group of 122 FNAC preparations, 79 patients could have avoided an unnecessary surgical intervention if the decision about the operation had been based on the results of molecular testing. This finding corresponds to a potential ~14-fold decrease in the number of unnecessary surgeries [81]. 

In order to organize molecular biomarker diagnosis, to become not only used in clinical studies but also available for all patients, NGS must become available and widely commercial, however, it is highly expensive. In this form we will encounter molecular panels such as Afirma Gene Expression Classifier (GEC) from Veracyte Inc. San Francisco, CA, USA, which uses a 142 mRNA-based panel, ThyroSeq and ThyroSeq v2 (2011), and since 2017 ThyroSeq v3 has been available, which interrogates selected regions of 112 thyroid cancer-related genes for point mutations, insertions/deletions, gene fusions, copy number alterations, or gene expression alterations, and, also, Afirma Gene Sequencing Classifier (GSC). In May 2018, Veracyte Inc. launched the Afirma Xpression Atlas (XA) which uses RNA sequencing. Additionally, miRNA are commercially used: MPT, Interpace Diagnostics Parsippany, NJ, USA combines a mutation panel ThyGenX that interrogates nine hotspot regions in the BRAF, HRAS, KRAS, NRAS, and PIK3CA genes, six fusion transcripts, and a miRNA classifier test (ThyraMIR). RosettaGX Reveal (Rosetta Genomics North Brunswick, NJ, USA) is a thyroid miRNAs classifier that utilizes RNA extracted from Papanicolaou or Romanowsky-type (Diff-Quik and Giemsa) stained directly, or ThinPrep smears and has an algorithm based on a panel of 24 miRNAs to classify cytologically indeterminate thyroid nodules into benign, suspicious for malignancy or positive for MTC [83]. Statistical results from clinical validations studies are synthesized in Table 4.

Recent meta-analysis investigated studies that used newer NGS panels such as ThyroSeq v3 and Afirma (GSC) compared with old commercial panels. ThyroSeq v3’s sensitivity reached 93.4–100% and its specificity reached 16.7–100%. Afirma (GEC) had a sensitivity of 90.6–100% and a specificity of 28.6–68.3% in accordance with a previously published meta-analysis conducted by Liu et al. which found a sensitivity of 95.5% and a specificity of 22.1% with NPV at 88.2% and PPV at 44.3% [89]. The number of GSC negative results increased extensively to 72% compared with GEC, a fact also shown by Polavarapu et al., who also found a surgical rate decrease to 40% in GSC compared to 59% in GEC, and a malignancy rate that increased to 39% for GSC compared to 22% for GEC [90]. The ThyGenX oncogene panel had a sensitivity of 94.3% and a specificity of 61.4%. RosettaGX Reveal had a sensitivity of 85.2 to 100% and a specificity of 69.2 to 85.7% across studies. ThyraMir, less used than others, completed a sensitivity of 50% and a specificity of 91.9% as reported by Silaghi et al. [83]. What is clearly seen from these multiple studies is that PPV increased considerably for Afirma GSC compared to GEC and ThyroSeq v3 had better PPV than ThyroSeq v2. [83]. All these panels concur with a personalized diagnosis in favor of precision medicine that is involved in thyroid carcinoma diagnosis and treatment as seen in Table 5. 

Personalized diagnosis includes, also, particular territories, such as Asian countries which are well known for thyroid carcinoma incidence that became a public health problem. NGS and miRNA results turn into personalized diagnoses in this particular situation. The influence of molecular tests on the treatment of indeterminate nodules was determined in another meta-analysis that examined the differences between Western and Asian countries. They found that, at the rate and malignancy risk for indeterminate nodules, among the users of Afirma GEC, ThyroSeq and single-gene testing (e.g., BRAF V600E), had some discordant results: Afirma GEC’s resection rate was 46.6% vs. risk of malignancy 37.4%, ThyroSeq’s resection rate was 35.4% vs. risk of malignancy 21.3%, and BRAF’s rate of resection was 39.7% vs. malignancy rate of 60.7% [96]. Single gene testing, BRAF V600E, proved great accuracy in diagnosing thyroid carcinoma and also, a good decrease of thyroid surgeries due to single-gene positive results meaning that only one gene detection could predict ROM as well as NGS panels. 

The diagnostic value of circulating miRNAs for PTC was determined in another meta-analysis which concluded that miR-222 and miR-146b may be prime candidates for the diagnosis of PTC in an Asian population, and NGS of miRNA has a sensitivity of 79% and specificity of 82% to diagnose thyroid carcinoma [97]. 

A new framework comes from liquid biopsy, which could reduce at minimum reanalysis of thyroid biopsies in cases of Bethesda I, nondiagnostic from lack of proper cellular material, or even for indeterminate cytology. To determine DNA or miRNA, only a few nanograms of biological material is required. So, this is very accessible for thyroid aspiration procedures, in which, in some patients, clinicians procure insignificant cytological material. From the same needle puncture, cytological smears are collected and liquid biopsy is prepared by rinsing the needle for remaining material with 2–10 mL of saline water, then immediately centrifuged to obtain cell pellets, which could be processed either as a cytospin preparation or as a formalin-fixed, paraffin-embedded cell block. The supernatant will be stored at 4 °C until the cytological diagnosis will be finalized and then the DNA extraction and subsequent molecular testing could begin. Ye et al. found that 156 cytological samples yielded adequate DNA (median, 135 ng; range, 11–3180 ng), and 129 of these samples (83%) were successfully sequenced by NGS. The most frequently detected somatic mutations included BRAF and RAS mutations, which were followed by RET, TP53, PTEN, CDKN2A, and PIK3CA mutations. A total of 11 of 31 cases with Bethesda III diagnosis, and 9 of 12 with Bethesda IV diagnosis, had somatic mutations, including BRAF V600E, which is highly definitive for PTC. A total of 7 of the 9 patients with positive BRAF V600E had a surgical follow-up, and they were all confirmed to have PTC. A comparison of the mutation profiles, in a small subset of cases (8 patients), showed that mutational results derived from supernatants, paired smears, and/or cell blocks, and had 100% concordance in all above-used methods [98].

A dual approach using both genotypic and phenotypic tests, NGS, and miRNA expression-based tests (e.g., Thy-GeNEXT and ThyGenX) must be used to best characterize and predict the status of malignancy risk of each individual thyroid nodule. This approach, ThyGeNEXT, increased the positive cases of malignancy and decreases the negative results compared to ThyGenX. Combined, these two panels will improve diagnostic accuracy and surgeries will be in decline [99].

Comparative data were made for use in the real world Afirma GEC and ThyroSeq v2 panels, along with already published results. Some inconveniences consist in the discrepancies between NPV and PPV: real-world NPV was 85–90%, compared with 95% NPV in the study results. For PPV, real-world results were 20–40% compared to studies where PPV was 40–90% [100]. Real-world NGS results were inferior to those from published data, meaning that double-check analysis criteria are needed when using molecular biomarkers in our day-by-day patients. 

Recent research suggests that American Thyroid Association ultrasound criteria may have similar, slightly inferior diagnostic performance, compared to Afirma GEC. The combination of data from ultrasound and Afirma GEC may alter the performance of GEC for the diagnosis of indeterminate nodules. They propose that in circumstances in which molecular testing is not available, ultrasound risk assessment may be used to risk-stratify indeterminate nodules [101]. 

## 5. Artificial Intelligence in Thyroid Carcinoma

AI debuted in order to help the precise diagnosis, facilitating clinicians’ work. AI started using thyroid nodules imaging characteristics, that form a pattern, and this pattern was being transformed into AI feedback for future explored nodules. AI, used for the diagnosis of thyroid nodules, has a major intention for differentiating malignant thyroid nodules from benign ones. Machine learning (ML) features, where the computer is trained to perform tasks by learning from example data and make predictions based on its exposition to previous samples are used for this purpose, and many studies implemented ML to facilitate the computerized diagnosis approach. Peng et al. developed a deep learning (DL) AI model (ThyNet) to differentiate malignant from benign thyroid nodules. The major investigation was if radiologists could improve their diagnostic performances, with the assistance of the ThyNet model, when reading ultrasound images and videos. ThyNet explored the potential of the assisted strategy to help radiologists/clinicians avoid unnecessary fine needle aspirations. Accuracy diagnosis for DL machinery was better than radiologists interpreting those images. FNAC decreased dramatically from 61.9% to 35.2%, in this study using the ThyNet assisted strategy, while missed malignancy results slightly decreased from 18.9% to 17.0% [102]. 

DL is a new classification of ML, meaning that DL relies on networks of computational units, neural units arranged in layers that gradually extract higher-level features from input data and images. These structures learn discriminative features from data automatically, allowing them to approximate complex nonlinear relationships with outstanding performance. DL is able to achieve diagnosis computerization avoiding human intervention (Table 6) [103]. 

Park et al. used DL in a prospective study when thyroid nodules were analyzed by a DL machine. Ultrasound images were compared to radiologists’ interpretations. Results were similar for DL and radiologist interpretations considering sensitivity, specificity, and PPV. DL machine improved PPV for inexperienced radiologists, a plus for residents that are just beginning to interpret thyroid nodules features. Overall, radiologists had lower misclassified malignancy cases (9 cases) than DL (12 cases), which is interpreted by radiologists with vast experience in analyzing thyroid nodules details [104].

**Table 6:** Summarizing the accuracy of deep learning results from recently published studies [105,106,107,108,109,110,111,112,113,114].

	Accuracy	Sensitivity	Specificity
Kim et al. 2021	85.10%	81.80%	86.10%
Wu et al. 2021	82%	85%	78%
Jin et al. 2020	80.30%	80.60%	80.10%
Liang et al. 2020	75%	84.90%	69%
Buda et al. 2019	N/A	87%	52%
Ko et al. 2019	87.30%	90%	82%
Park et al. 2019	86%	91%	80%
Wang et al. 2019	90.30%	90.50%	89.90%
Li et al. 2019	86%	84%	87%
Chi et al. 2017	96.30%	82.80%	99.30%
Ma et al. 2017	83%	82.40%	84.90%

N/A—not assessed.

DL was used for thyroid scintigrams, to prove that a machine could also learn the thyroid uptake pattern. Results were the following for sensitivity: 90.77% (236/260 patients) for the “diffusely increased” pattern, 99.56% (225/226) for “diffusely decreased”, 100.00% (25/25) for “focal increased” in the internal validation, whereas the recall for “heterogeneous uptake” was relatively moderate at 81.48% (88/108). The model proposed by this study (ResNet50, DenseNet169, InceptionV3, and InceptionResNetV2) had high advantages in the recognition of “diffusely increased” and “diffusely decreased” patterns [115]. 

**Figure 5:**
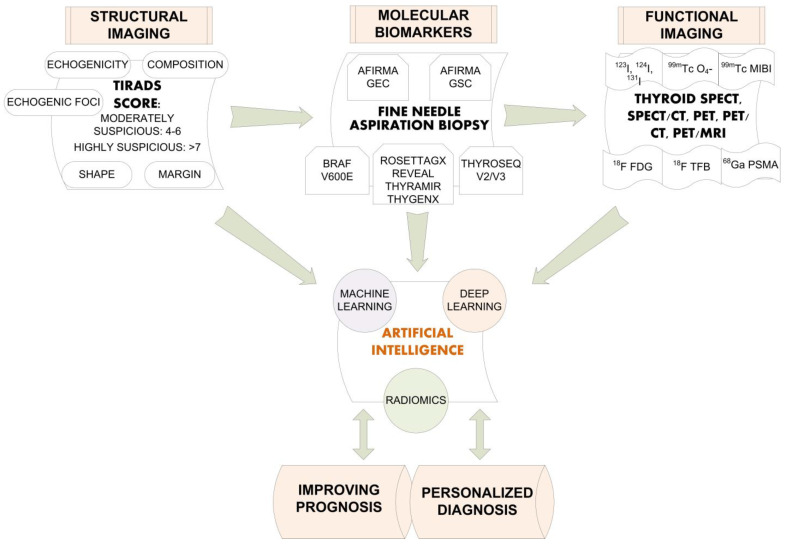
Proposed algorithm for the diagnosis of differentiated thyroid carcinoma.

AI could improve thyroid nodules diagnosis, reaching for an accurate imaging result, as seen by the summarized accuracy data of recently published studies (Table 6). With a precise learned pattern, AI could tell, only by imaging features, if thyroid nodules are malignant or benign. For future perspectives, AI could have better availability and could be accessible day by day for clinicians’ practice and imagery specialists (Figure 5). 

## 6. Conclusions

In order to find a personalized diagnosis for our patients, we have to know the available diagnostic tools; therefore, this review highlights quantitative functional imaging and molecular biomarkers as the first steps in artificial intelligence algorithms. 

The hard work comes when FNAC results are indeterminate: Bethesda III and IV. In this situation molecular biomarkers are very useful with more NGS panels developed for commercial use purposes and future gene expression classifiers, that will comprise even more DNA and RNA dysfunction diagnosis on thyroid tissue or even simpler in FNAC. Additionally, radioiodine refractory thyroid cancers are not easy to manage, so in this situation comes in hand, not only different imagistic investigation as SPECT, PET, PET/CT/MRI with different radiotracers, but also new targeted therapies for thyroid carcinoma could improve patient management. Over the years, we are seeing an increasing trend in iodine refractory metastatic disease. The availability of ^18^F FDG PET/CT, and many more SPECT or PET radiotracers imaging, has helped in the early detection of these lesions, improving multimodality management protocols.

PRRT in thyroid carcinoma is a future perspective for DTC and more prospective trials will determine if radioiodine refractory thyroid disease will be managed by this kind of treatment. Targeted gene therapy is in continuous development, but what we need is the availability of specific medication day by day, not only in clinical trials. Known facts about DTC are sometimes contradictory or mixed with only theoretical thoughts. Guidelines that should permit the implementation of an updated management strategy that is based on scientific evidence will be well deserved by all of us clinicians when dealing with difficult decisions for our patients.

## Figures and Tables

**Table 1:** Prevalence and risk of malignancy of ^18^F FDG PET and PET/CT thyroid incidentalomas according to published meta-analyses (adapted from Piccardo A et al. Rev Endocr Metab Disord. 2019 [21,22].

Authors (Publication Year)	Number of Studies	Number of Patients	Pooled Prevalence (95%CI)	Pooled Risk of Malignancy (95%CI)
Shie et al. (2009) [23]	18	55,160	1%	33.2%
Soelberg et al. (2012) [24]	22	125,754	1.6%	34.8%
Bertagna et al. (2012) [25]	27	147,505	2.46% (1.68–3.39)	34.6% (29.3–40.2)
Treglia et al. (2013) [26]	34	215,057	1.92% (1.87–1.99)	36.2% (33.8–38.6)
Qu et al. (2014) [27]	29	196,298	2.9%	NR
Nayan et al. (2014) [28]	31	197,296	1.9%	20% (15.3–25)
**de Leijer JF et al.** **(2021) [29]**	**61**	**660,037**	**2.22%**	**12.6%**
**Scappaticcio L et al. (2021) [30]**	**15**	**2304**	**NR**	**19% (13–25)**

CI—confidence interval; NR—not reported.

**Table 2:** The ability of ^18^F FDG PET/CT to detect thyroid malignancy in patients with indeterminate thyroid nodules (adapted from Piccardo A et al. Rev Endocr Metab Disord. 2019) [21].

Authors (Publication Year)	Number of Studies	Number of Patients	Prevalence of Malignancy	Sensitivity (95%CI)	Specificity (95%CI)	NPV (95%CI)	PPV (95%CI)
Wang et al. (2013) [31]	7	267	26.2% (19.6–40)	89% (79–95)	55% (48–62)	NR	[1]NR
Vriens et al. (2014) [32]	6	225	25.8% (13.6–41.7)	95% (86–99)	48% (40–56)	96% (90–99)	39% (31–47)
**Castellana M. et al. (2019) [33]**	**8**	**431**	**NR**	**74% (58–84)**	**58% (48–67)**	**74% (41–100)**	**34% (25–44)**
**Qichang et al. (2019) [34]**	**13**	**634**	**24% (4–50)**	**63%**	**65%**	**55%**	**44%**

CI—confidence interval; NR—not reported, NPV—negative predictive value, PPV—positive predictive value.

**Table 3:** Diagnostic performance of ^18^F FDG PET/CT in DTC restaging according to published meta-analyses (adapted from Piccardo A et al. Rev Endocr Metab Disord. 2019) [21].

Authors (Publication Year)	Number of Studies	Number of Patients	Sensitivity (95%CI)	Specificity (95%CI)
Dong et al. (2009) [35]	6	165	93% (87–97)	84% (72–92)
Miller et al. (2011) * [36]	11	498	82% (69–94)	84% (77–92)
Caetano et al. (2016) [37]	7	260	93% (84–97)	81% (69–90)
Haslerud et al. (2016) [38]	17	905	80% (73–86)	75% (63–85)
Schütz et al. (2018) [39]	11	NR	94% (87–98)	78% (52–92)
Kim et al. (2018) [40]	9	515	84% (77–89)	78% (67–86)
**Qichang et al. (2019) [41]**	**17**	**1195**	**86%**	**84%**

CI—confidence interval; NR—not reported; * both PET and PET/CT.

**Table 4:** Clinical validation studies results of commercially NGS used in patients with indeterminate FNAC (adapted from Agarwal et al. [84]).

	Afirma Gene Sequencing Classifier (GSC) [85]	ThyroSeq v3 [86]	RosettaGX Reveal [87]	ThyraMir [88]
Sensitivity	91%	94%	74%	89%
Specificity	68%	82%	74%	85%
Negative predictive value	96%	97%	94%	92%
Positive predictive value	47%	66%	43%	74%

**Table 5:** Molecular panels diagnosing thyroid malignancy in patients with indeterminate thyroid nodules, a summary of published meta-analysis.

	Author/Year	No. of Studies	No. of Thyroid Nodules	Sensitivity	Specificity	Negative Predictive Value	Positive Predictive Value
**Afirma Gene** **Expression Classifier (GEC)**	Sciacchitano et al. 2017 [91]	2	210	90%	52%	94%	37%
	Borowczyk et al. 2018 [92]	16	1086	98%	12%	91%	45%
	Vargas-Salas et al. 2018 [93]	19	1371	92%	27%	91%	30%
	Liu et al. 2019 [89]	18	3290	95.5%	22.1%	88.2%	44.3%
	Vuong et al. 2020 [94]	7	1947	93.6%	25.1%	86.1%	41.6%
	Silaghi et al. 2021 [83]	25	4538	97%	19%	91%	39%
**Afirma Gene Sequencing Classifier (GSC)**	Vuong et al. 2020 [94]	7	807	94.3%	43%	90%	63.1%
	Silaghi et al. 2021 [83]	4	635	95%	51%	91%	60%
**ThyroSeq v2**	Borowczyk et al. 2018 [92]	5	459	84%	78%	93%	58%
	Vargas-Salas et al. 2018 [93]	5	350	86%	79%	94%	58%
	Sciacchitano et al. 2017 [91]	9	143	90%	93%	96%	83%
	Silaghi et al. 2021 [83]	9	1549	86%	75%	95%	51%
**ThyroSeq** **v3**	Silaghi et al. 2021 [83]	4	603	99%	64%	96%	78%
**ThyGenX**	Sciacchitano et al. 2017 [91]	8	1141	51%	93%	86%	70%
	Vargas-Salas et al. 2018 [93]	1	109	89%	85%	96%	66%
	Silaghi et al. 2021 [83]	3	141	94.3%	61.4%	N/A	N/A
**RosettaGX Reveal**	Vargas-Salas et al. 2018 [93]	1	150	74%	74%	90%	49
	Silaghi et al. 2021 [83]	3	234	85.2–100%	69.2–85.7%	N/A	N/A
**ThyraMir**	Sciacchitano et al. 2017 [91]	2	109	89%	85%	94%	74%
	Silaghi et al. 2021 [83]	1	105	50%	91.9%	N/A	N/A
**BRAF V600E**	Sciacchitano et al. 2017 [91]	24	2625	41%	100%	68%	99%
	Fnais et al. 2015 [95]	9	262	52%	100%	N/A	N/A

N/A—not assessed.
